# Hopelessness and Excessive Drinking among Aboriginal Adolescents: The Mediating Roles of Depressive Symptoms and Drinking to Cope

**DOI:** 10.1155/2011/970169

**Published:** 2010-10-11

**Authors:** Sherry H. Stewart, Simon B. Sherry, M. Nancy Comeau, Christopher J. Mushquash, Pamela Collins, Hendricus Van Wilgenburg

**Affiliations:** ^1^Department of Psychiatry, QEII Health Sciences Centre, Dalhousie University, 5909 Veteran's Memorial Lane, 8th floor Abbie J. Lane Memorial Building, Halifax, NS, Canada B3H 2E2; ^2^Department of Psychology, Life Sciences Centre, Dalhousie University, 1355 Oxford Street, Halifax, NS, Canada B3H 4J1; ^3^School for Resource and Environmental Studies, Dalhousie University, Kenneth C. Rowe Management Building, 6100 University Avenue, Suite 5010, Halifax, NS, Canada B3H 3J5

## Abstract

Canadian Aboriginal youth show high rates of excessive drinking, hopelessness, and depressive symptoms. We propose that Aboriginal adolescents with higher levels of hopelessness are more susceptible to depressive symptoms, which in turn predispose them to drinking to cope—which ultimately puts them at risk for excessive drinking. Adolescent drinkers (*n* = 551; 52% boys; mean age = 15.9 years) from 10 Canadian schools completed a survey consisting of the substance use risk profile scale (hopelessness), the brief symptom inventory (depressive symptoms), the drinking motives questionnaire—revised (drinking to cope), and quantity, frequency, and binge measures of excessive drinking. Structural equation modeling demonstrated the excellent fit of a model linking hopelessness to excessive drinking indirectly via depressive symptoms and drinking to cope. Bootstrapping indicated that this indirect effect was significant. Both depressive symptoms and drinking to cope should be intervention targets to prevent/decrease excessive drinking among Aboriginal youth high in hopelessness.

## 1. Introduction

Alcohol misuse is a serious problem among many North American Indigenous communities [1]. In Canada, the Aboriginal^1^ Peoples Survey [2] showed that 73% of First Nations respondents reported that alcohol was a problem in their communities. A recent review concluded that rates of alcohol misuse are higher among American Indians than among those in the general U.S. population, and that this is true for both adults and adolescents [3]. For example, rates of past month drunkenness are about twice as high among Native American adolescents as among nonnative American adolescents [3]. The 2002-03 First Nations Regional Longitudinal Health Survey [4] suggests this is also true for Canadian Aboriginal people, for example, the proportion of Aboriginals who reported weekly heavy drinking (5+ drinks on a single occasion) was more than double that of those in the general Canadian population (16.0% versus 7.9%, resp.), despite the fact that when compared to the general Canadian population, Aboriginals are less likely to be current drinkers [5]. Some research suggests that Aboriginal youth may be particularly susceptible to excessive drinking [6].

These high rates of excessive drinking have many negative consequences for Aboriginal communities. For example, death related to alcohol use disorders is higher for Aboriginal people than for other ethnic groups [7]. In fact, alcohol has been identified as a leading case of adolescent morbidity and mortality consequent to violence, falls, suicide, drowning, motorvehicle accidents, and risky sexual behavior [8]. For these reasons excessive drinking among Aboriginal youth must be considered an important public health concern [9].

In addition to elevated rates of excessive drinking, other problems faced by Aboriginal youth include high levels of depressive symptoms (e.g., extreme sadness, loss of interest, suicidality, and fatigue) and hopelessness (i.e., feelings of worthlessness and pessimism about the future). Profound changes brought upon Aboriginal peoples through colonialism have been linked to high rates of depressive symptoms and suicide in many communities [10]. Examples of such profound changes include geographic dislocation and disruptions to their connections to the land and to their traditional patterns of subsistence [1]. Depressive symptoms and suicide appear to be particular problems for Aboriginal youth [11]. Discrimination has been associated with depressive symptoms in both American Indians adults [12] and adolescents [13]. Abject poverty created by colonial policies has resulted in a lack of control for Canadian Aboriginal peoples, which has contributed to feelings of hopelessness [6, 14]. 

Cognitive theories of depression posit a key role to hopelessness as an individual differences factor that sets the stage for the development of depressive symptoms (e.g., [15, 16]). While it is well established that hopelessness is a risk factor for depression in nonAboriginal groups (e.g., [17–19]), there is little data examining the impact of hopelessness on depressive symptoms among Aboriginal peoples. One cannot simply assume that hopelessness leads to a given mental health outcome in the same way in Aboriginal communities as it does in nonAboriginal groups (e.g., see [20]).

It is well established in nonAboriginal people, that depression and alcohol abuse/dependence commonly cooccur (see reviews in [21–23]). American epidemiologic surveys in the adult general population [24, 25] show significant odds ratios for depression comorbid with alcohol use disorders indicating that the two disorders cooccur at rates that far exceed chance. A number of studies have confirmed a cooccurrence of depression and alcohol misuse in nonAboriginal adolescents as well (see review in [23]). For example, it has been shown that depression rates increase from about 5% in American youth who abstain from alcohol to about 24% in those who use alcohol at least weekly [26]. 

In nonAboriginals, hopelessness has been linked to alcohol use and abuse in both adults and adolescents (e.g., [18, 19]), for example, a cross-sectional study found that hopelessness in adolescents was significantly positively related to lifetime alcohol use [27]. It was also shown that hopelessness was associated longitudinally with alcohol use and drunkenness over a one-year interval in a group of Canadian adolescents [28]. In sum, there are clearly established relations between hopelessness and depression with excessive drinking in nonAboriginal youth. Data that directly examines and tests the relationships between hopelessness, depressive symptoms, and excessive drinking is lacking, however, among Aboriginal adolescents.

A number of theoretical models have been proposed to explain the relation between depression and alcohol misuse (see reviews in [23, 29]). One popular model is *self-medication* [30], which posits that depressed individuals drink to reduce negative emotions, and are thus at risk for heavier drinking and more alcohol-related problems as a consequence. Consistent with this theory, elevated depressive symptoms in adolescence have been shown to predict future alcohol use disorders (e.g., [31]), alcohol-related problems (e.g., [32]), and alcohol use levels (e.g., [33]), for example, in a study of 1545 Finnish twins, it was found that early onset depressive disorder (at age 14 years) predicted later alcohol use and recurrent intoxication (at age 17.5 years), even after the effects of other substance use and other psychiatric disorders were controlled [34]. 

Few studies, however, have tested the underlying mechanism posited in self-medication theory—namely, drinking-to-cope with negative emotions. In two separate groups of nonAboriginal Canadian adolescents, it was shown that consistent with self-medication theory predictions, drinking to cope mediated the relations of both hopelessness and depressive symptoms with alcohol-related problems [35]. It was concluded that both hopelessness and depressive symptoms play a role in adolescent motivation for alcohol use, with both reflecting a desire to diminish negative affect [35]. However, the study did not test the possibility that hopelessness might be related to drinking to cope indirectly via its effects on depressive symptoms. 

**Figure 1:**
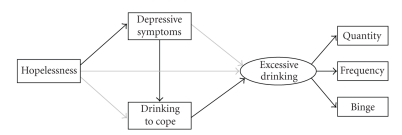
The hypothesized structural model. Rectangles represent manifest variables; ovals represent latent variables. Black arrows represent hypothesized direct effects; grey arrows represent paths hypothesized to be explained by indirect effects. Quantity = drinking quantity; Frequency = drinking frequency; Binge = binge drinking.

The purpose of the present study was to examine the relations among four variables (i.e., hopelessness, depressive symptoms, drinking to cope, and excessive drinking) assessed cross-sectionally in a large group of Canadian Aboriginal adolescent drinkers. We propose that Aboriginal adolescents with higher levels of hopelessness are more susceptible to depressive symptoms, which in turn predispose them to drinking to cope—a drinking motivation that puts them at greater risk for excessive drinking. Thus, we specified a structural equation model in which we hypothesized (a) hopelessness would be directly linked to depressive symptoms, (b) depressive symptoms would be directly linked to drinking to cope, and (c) drinking to cope would be directly linked to excessive drinking. We also specified three meditational hypotheses. We expected (d) hopelessness to be indirectly related to drinking to cope through depressive symptoms, (e) depressive symptoms to be indirectly related to excessive drinking through drinking to cope, and (f) hopelessness to be indirectly related to excessive drinking through depressive symptoms and drinking to cope. The hypothesized structural model is depicted in Figure 1.

## 2. Materials and Methods

### 2.1. Participants

Participants were drawn from among 837 student respondents enrolled in any one of 10 participating schools in the Canadian provinces of Saskatchewan, Manitoba, and Quebec. All participating schools contained a large representation of Aboriginal students. Six schools were from the Canadian province of Saskatchewan, two from Manitoba, and two from Quebec (one from a Cree and one from an Inuit community). Both urban (*n* = 2) and rural (*n* = 8) schools were represented. Of the total 837 students, 286 (34%) indicated that they had not consumed any alcohol in the last four months, leaving 551 participants (66%) to be classified as “drinkers” (52% boys; mean age 15.9 yrs., SD = 1.3, range = 14–18; mean grade = 9.4, SD = 1.2, range = 7–12). Of the 551 drinkers, 178 (32%) self-identified as Cree, 91 (16%) as Ojibway, 57 (10%) as Metis, 32 (6%) as Oji-Cree, and 25 (5%) as Dakota. A further 29 (5%) were classified as “Other Aboriginal”.^2^ Also included among the drinkers, due to their attendance at the participating schools, were 63 (12%) students who self-identified as Caucasian or Black.^3^ A further 76 (14%) drinkers skipped the race/ethnicity question altogether.^4^ Of the 551 drinkers, 326 (59%) were from schools in Saskatchewan, 168 (31%) from schools in Manitoba, and 57 (10%) from schools in Quebec. Only the data provided by the 551 drinkers were employed in all subsequent analyses. Abstainers were excluded from completing the measure of drinking to cope because the measure used requires that respondents have at least some relatively recent drinking experience to answer items enquiring about why they drink. The inclusion of even infrequent, light drinkers (e.g., those having consumed only one drink in the last four months) ensured that the results would be applicable to a wide range of adolescent drinkers from participating schools (see [36]).

### 2.2. Measures

#### 2.2.1. Substance Use Risk Profile Scale (SURPS)

The SURPS [19] is a 23-item self-report scale designed to measure four individual difference factors, including hopelessness, each of which has been shown to be related to risk for alcohol use/misuse. Participants are asked to rate the degree to which they agree with each item on a scale ranging from 1 = strongly disagree to 4 = strongly agree. A sample hopelessness scale item is “I feel that I am a failure.” The SURPS hopelessness score is calculated by summing across all 7 hopelessness items following reverse scoring of several inverselykeyed items. The SURPS has been shown to have a stable four factor structure and the four scales have been found to have good internal consistency and good construct validity in nonclinical adolescents recruited through Canadian schools [19]. In particular, the hopelessness scale shows significant bivariate correlations with depressive symptoms, drinking to cope, drinking quantity, drinking frequency, binge drinking, and alcohol-related problems in nonAboriginal Canadian youth [19]. The SURPS hopelessness scale has also been shown to correlate significantly with lifetime alcohol involvement in a group of American adolescents [27]. In the present study, the internal consistency of the hopelessness scale was acceptable at *α* = .78.

#### 2.2.2. Depression Subscale of the Brief Symptom Inventory (BSI-DEP)

The BSI-DEP [37] is a 7-item measure that assesses depressive symptoms. The full BSI was developed as a brief version of its longer parent instrument, the 90-item Symptom Check List—Revised (SCL-90-R; [38]. Each item is rated on a scale ranging from 0 = not at all to 4 = extremely. A sample BSI-DEP item is “Feeling no interest in things.” The BSI-DEP total score was calculated by summing across all seven depression items. Factor analytic studies of the BSI suggest good structural validity (e.g., all BSI-DEP items show salient loading on a single depression factor). Both test-retest reliability and internal consistency are high for the BSI-DEP scale and it correlates well with the depression scale from the original SCL-90-R. Moreover, the BSI-DEP scale shows high convergence with other established depression scales [37]. While there are many validated measures of depression for use in adolescents, we chose the BSI-DEP scale mainly for its brief length (i.e., 7 items) in order to reduce participant burden. In the present study, the internal consistency of the BSI-DEP was good at *α* = .86.

#### 2.2.3. Drinking Motives Questionnaire—Revised (DMQ-R)

The DMQ-R [39] is a 20-item self-report measure that taps four distinct motivations for alcohol use among adolescents, including drinking to cope. Respondents indicate their relative frequency of alcohol use in each of the indicated circumstances, when they drink. Each subscale consists of five items which are rated on a scale ranging from 1 = almost never/never to 5 = almost always/always. Subscale scores are computed as the mean of the relative frequency ratings for each of the five items on each subscale [39–41]. The drinking to cope scale measures drinking to reduce or avoid a range of negative affective states and consists of items such as “Because it helps you when you feel depressed or nervous.” Prior work shows that the drinking to cope scale is associated both with excessive drinking and with adverse consequences of drinking among groups of American, Swiss, and Canadian adolescents [39, 42]. The drinking to cope scale has also been shown to have good internal consistency and structural validity among Canadian Aboriginal adolescents [43]. In the present study, the internal consistency of the drinking to cope scale was good at *α* = .81.

#### 2.2.4. Excessive Drinking

Excessive drinking was indexed with three items assessing degree of alcohol use in the last four months (see also [19]). First, participants indicated the number of alcoholic beverages they normally consumed per drinking day; this index was referred to as drinking *quantity*. Participants were informed with visual and verbal cues that one drink equals one bottle/can of beer, one small glass of wine, one shot of hard liquor, or one cooler. Next, participants reported how often they normally consumed alcohol; this index was referred to as drinking *frequency*. Finally, participants indicated how often they normally consumed 5+ drinks in a single sitting (4+ drinks for girls); this index was referred to as *binge* drinking. Each item was rated on a scale of 1–5, such that high scores on each of the three measures index higher levels of *excessive* (i.e., more frequent, heavy, or intense) drinking. Response options for the quantity item were 1 = 1 or 2, 2 = 3 or 4, 3 = 5 or 6, 4 = 7 to 9, and 5 = 10 or more. Response options for the frequency item were 1 = less than monthly, 2 = once per month, 3 = 2 to 3 times per month, 4 = weekly, and 5 = daily or almost daily. Response options for the binge item were 1 = never, 2 = less than monthly, 3 = monthly, 4 = weekly, and 5 = daily or almost daily. Previous research has found adequate reliability of self-reported alcohol consumption measures across a broad range of response formats [44]. Nonetheless, recommended methods were used to enhance the accuracy of participants' self-reports [45]. Specifically, drinking behavior items were embedded within other questions on demographics to minimize their salience. Moreover, since extensive evidence supports the validity of self-reported alcohol use when participants are assured confidentiality [46], students were verbally assured confidentiality prior to survey completion. In the present study, this three item measure showed acceptable internal consistency (*α* = .79).

### 2.3. Procedure

This study was part of a larger project on alcohol abuse prevention in the 10 participating schools. The project received approval from the Dalhousie Health Sciences Human Research Ethics Board (protocol no.: 2007-1628) and Health Canada's Research Ethics Board (protocol no.: 2007-0026). Recruitment occurred through the active process of relationship- and partnership-building with the communities involved. Policing partners and other community members (e.g., Elders) approached the investigators upon learning of our previous alcohol abuse prevention work in Aboriginal (i.e., Mi'kmaq) communities in Nova Scotia [47]. Essentially, community partners self-identified for inclusion in the larger project. Community partners then identified schools that would be interested in being involved. This study engaged Aboriginal youth (grades 7–12) through its grounding in the school system of the Aboriginal community. Reflecting the deep value of Elders' knowledge of the participating communities, the project was arranged to encourage meaningful participation among school partners, policing partners, and study investigators.

School administration partners advised as to the method of distributing information about the study to parents/guardians of students in grades 7–12 in participating schools, prior to administration of the survey. An information sheet describing the study was sent in a mail-out directly to parents/guardians. Parents/guardians were encouraged to contact the researchers or school principal for any further information they desired about the study. Parents/guardians were asked to let the researchers/school principal know if they did not consent to having their child participate (i.e., a negative consent procedure was used). Parents/guardians were provided with a toll-free number to contact the researchers for additional information, if they so desired. 

School administration partners at each site also advised as to whether announcements describing the study should be delivered school-wide through the loud-speaker system along with regular morning announcements and/or delivered by individual classroom teachers. Prior to survey administration, students were informed about the nature of the study, and willing students provided written informed consent at the time of the survey. Consent forms were maintained separately from the completed questionnaires to ensure confidentiality and anonymity. Students were informed that the purpose of the survey was to investigate individual differences in reasons for alcohol use. 

The student consent form provided potential participants with information about the purpose of the survey, as well as the voluntary and confidential nature of the questions. Students were told that they were free to decline to participate and free to withdraw at any time. Those who declined participation were invited to the school library (under the librarian's supervision) or were asked to remain seated and read while their classmates were completing the survey. All students in grades 7–12 in participating schools were invited to take part in the survey. Approximately 20 students in total declined study participation resulting in a very high response rate (approximately 98% from among eligible students who were attending school on the day of survey administration). Data collection was conducted on a grade-by-grade basis during class time, with the permission and input of the school principal. Data collection was led by one of the coauthors (MNC). Following survey administration, the researcher leading the data collection offered a brief presentation on psychology research. No feedback was given to parents, teachers, or students regarding individual students' scores. Teachers had the option of remaining in the classroom at the time of the survey. Measures were administered in a standard order as follows: demographics, excessive drinking indices, BSI-DEP, DMQ-R, and SURPS. During questionnaire completion, students were permitted to ask questions of the researchers. The small minority of students who had difficulties with reading were offered assistance in reading the survey questions by trusted teachers or classroom aides. Translation into French was provided for the students in the Cree school in Quebec.

In order to protect any student from being singled out and labeled, the survey was anonymous. To maintain anonymity and confidentiality, students were asked not to write their names on the forms. The toll-free number mentioned above that was established for parent communication was also offered to students in case they had had any questions about the survey forms in particular, or about the research project more generally. The privacy of each call was ensured because one of the coauthors (M. N. Comeau) was the only person who took the calls. Although participants were not compensated financially, survey administrations were concluded with a snack or meal. By integrating an educational component into data collection and engaging the students, they had the opportunity to participate in a project where the ultimate goal was to develop future culturally relevant alcohol abuse prevention efforts that are more meaningful to the lives of youth in their communities.

## 3. Results

### 3.1. Descriptive Statistics and Bivariate Correlations

Mean (and SD) scores on each of the study measures for the total group of 551 drinkers are displayed in the right hand column of Table 1. The descriptive statistics for the three excessive drinking indices suggest that the average student was drinking relatively frequently, heavily, and intensely (i.e., drinking 2 to 3 times per month, consuming 5 or 6 standard drinks on each drinking occasion, and binge drinking monthly).

Bivariate correlations between the various study measures are also shown in Table 1. All study measures were significantly intercorrelated with one exception: depressive symptoms were not significantly correlated with drinking quantity. Several of the correlations among study variables were moderate to strong. However, multicollinearity and redundancy of variables are only a concern when correlations exceed 90 [48]. While the data in Table 1 suggests some expected overlap between several of the study variables (e.g., 24%–53% shared variance between the three indices of excessive drinking), none of the variables should be considered redundant. It is important to note that the correlation between hopelessness and depressive symptoms was significant (*r* = .42, *P* < .01) but did not approach the strength at which there would be concern about multicollinearity or redundancy. This result lends support to our conceptualization of hopelessness and depressive symptoms as distinct constructs.^5^

### 3.2. Structural Equation Modeling (SEM)

SEM was conducted using AMOS 7.0 [49]. Full information maximum likelihood estimation was utilized to deal with missing data [50]. For the structural model, fit was evaluated via multiple indices [51]. Adequate fit is indicated by a chi-square/degrees of freedom ratio (*χ* ^2^/*df*) around 2, a comparative fit index (CFI) and an incremental fit index (IFI) around .95, and a root-mean-square error of approximation (RMSEA) around .06 [52]. We report the RMSEA value along with 90% confidence intervals (90% CI).

**Figure 2:**
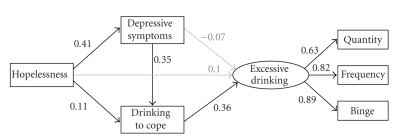
The hypothesized structural model. Rectangles represent manifest variables; ovals represent latent variables. Black arrows represent significant paths (i.e., *P* < .05). Grey arrows represent nonsignificant paths (i.e., *P* > .05). Path coefficients are standardized. Quantity = drinking quantity; Frequency = drinking frequency; Binge = binge drinking.

Three manifest variables were selected to represent the excessive drinking latent variable: drinking quantity, drinking frequency, and binge drinking. Each observed variable showed substantial and significant loadings (ranging from .63 to .89) on the excessive drinking latent variable. Fit indices also suggested the structural model fit the data well: *χ* ^2^(6, *N* = 551) = 12.93, *P* < .05; *χ* ^2^/*df* = 2.16; CFI = .99; IFI = .99; RMSEA = .05 (90% CI: .01,.08). The final model is depicted in Figure 2 with significant paths indicated with black arrows and nonsignificant paths indicated with grey arrows. As hypothesized, (a) hopelessness was directly linked to depressive symptoms, (b) depressive symptoms were directly linked to drinking to cope, and (c) drinking to cope was directly linked to excessive drinking (see Figure 2). These three above-mentioned direct paths were significant, substantial, and consistent with the hypothesized structural model (see Figure 1). Congruent with two of the mediational hypotheses, depressive symptoms were unrelated to excessive drinking, and hopelessness was unrelated to excessive drinking (see Figure 2). Unexpectedly though, hopelessness was directly linked to drinking to cope (*P* < .05; see Figure 2). It should be noted, however, that while the direct relationship between hopelessness and drinking to cope was significant, it was not much different in magnitude than the nonsignificant relationship between hopelessness and excessive drinking (see Figure 2).

A significant indirect effect indicates that mediation has taken place [53]. We used bootstrap analyses to test the significance level of the three hypothesized indirect effects (see Figure 1). For each test of indirect effects, we used random sampling replacement to create 20,000 (*n* = 551) bootstrap samples. These samples were then utilized to estimate bias-corrected standard errors for each hypothesized indirect effect in question. In the case of the indirect path from hopelessness to excessive drinking, the indirect effect was based on all paths and was computed by multiplying (a) path coefficients from the predictor to mediators and (b) path coefficients from mediators to the criterion. In addition, CIs were computed. An indirect effect may be described as significant (*P* < .05) when the 95% CI for this indirect effect does not include zero.

First, we tested the indirect effect of hopelessness on drinking to cope. Bootstrap estimates indicated this hypothesized indirect effect was significant: *β* = .143, *B* = .167, (95% CI: .094, .192), and SE = .025. That is, the indirect effect of hopelessness on drinking to cope through depressive symptoms was significant (see Table 2). Next, we tested the indirect effect of depressive symptoms on excessive drinking. Bootstrap estimates indicated this hypothesized indirect effect was also significant: *β* = .123, *B* = .019, (95% CI: .074, .173), and SE = .025. Put differently, the indirect effect of depressive symptoms on excessive drinking through drinking to cope was significant (see Table 2). Finally, we tested the indirect effect of hopelessness on excessive drinking. Bootstrap estimates indicated this hypothesized indirect effect was again significant: *β* = .059, *B* = .013, (95% CI: .002, .116), and SE = .029. That is, the indirect effect of hopelessness on excessive drinking through depressive symptoms and drinking to cope was significant (see Table 2).

In sum, results suggest the hypothesized structural model is a well-fitting model that is consistent with the expected pattern of direct and indirect effects in this group of Canadian Aboriginal adolescent drinkers (see also Endnote 5).

## 4. Discussion

Consistent with hypotheses, hopelessness was directly linked to depressive symptoms. This finding replicates, in an Aboriginal adolescent group, much previous work linking hopelessness with depression in nonAboriginal groups [17–19]. This is an important finding since hopelessness has not always been linked to mental health outcomes in Aboriginal groups in the same way it has been in nonAboriginal groups (e.g., [20]). The finding of a direct path from hopelessness to depressive symptoms within the structural model is consistent with predictions of models positing a key role for hopelessness as a cognitive risk factor for depression (e.g., [15, 16]). The discrimination, disruptions to family connections, geographic dislocation, and abject poverty arising from colonial policies have resulted in social and economic circumstances which are often objectively bleak for Canadian Aboriginal people, setting the stage for the development of hopelessness [6, 14]. But even Beck's model of depression acknowledges that such negative cognitions are not always distorted or inaccurate—merely that they are maladaptive in terms of increasing risk for depressive symptoms [54]. 

The present finding linking depressive symptoms directly to drinking to cope replicates, in Canadian Aboriginal adolescents, previous findings from nonAboriginal adolescents showing that depressive symptoms were related to drinking to cope with negative emotions [35]. The structural model also pointed to two ways in which hopelessness is linked to drinking to cope: the hypothesized indirect relation through depressive symptoms and an additional (unexpected) direct relation. This suggests that Aboriginal youth with higher levels of hopelessness are at increased risk of drinking to cope for two reasons. First, they are at risk of developing depressive symptoms which may motivate them to drink to eliminate or numb those unpleasant feelings. Second, hopeless individuals may attempt to block their pessimistic thoughts through drinking. It can be concluded, consistent with findings in nonAboriginal Canadian adolescents, that both hopelessness and depressive symptoms play a role in Aboriginal adolescents' motivations for alcohol use [35], with both reflecting a desire to diminish unpleasant cognitions or emotional states. 

The third hypothesized direct effect in the current study was a path from drinking to cope to excessive drinking. Cooper's model of adolescent drinking motives contends that drinking to cope is a particularly risky motivation for drinking that sets teens up for greater rates of excessive drinking and drinking-related problems [39]. The present finding showing a significant and substantial direct path between drinking to cope and excessive drinking in a Canadian Aboriginal group adds to the growing literature suggesting that the link between drinking to cope and excessive drinking persists cross-culturally [42]. 

As hypothesized, the relation of depressive symptoms to excessive drinking was indirect—an effect mediated through drinking to cope. This finding contributes to the understanding of the significant overlap between depression and alcohol use disorders in adults and adolescents alike [23] by suggesting one possible mechanism underlying this relationship. Specifically, the present findings suggest that adolescents with higher levels of depressive symptoms drink to excess more so than other adolescents because they are drinking to alleviate or numb negative emotions. This finding is consistent with the negative reinforcement mechanism postulated in self-medication theory [30] to explain the overlap of depressive symptoms and excessive drinking. The lack of a relation between depressive symptoms and drinking quantity in the bivariate correlations is, however, inconsistent with some previous research which has demonstrated such a link [55]. Nonetheless, this previous research was conducted with nonAboriginal adults rather than Aboriginal adolescents. 

Previous cross-sectional and longitudinal research with nonAboriginal youth suggests a relationship between hopelessness and excessive drinking [19, 27, 28]. Consistent with hypothesis, the path from hopelessness to excessive drinking in the present study was indirect—mediated through depressive symptoms and drinking to cope. It is interesting to consider this finding in relation to another model (alternative to the meditational model tested herein) that has been posited to account for the high overlap of depression and alcohol disorders—namely the common factors model [23, 29]. Specifically, it has been suggested that a third factor or common underlying vulnerability (such as hopelessness) contributes to the apparent association between depression and excessive drinking ([e.g., [17]). In other words, hopelessness is thought to independently and directly contribute to the development of both depressive symptoms and excessive drinking creating an apparent association between the latter two variables. The present findings are inconsistent with the common factors model given that we did not observe any direct relation between hopelessness and excessive drinking. Instead, the association between hopelessness and excessive drinking was indirect, and mediated through depressive symptoms and drinking to cope. 

Two comments should be made on the composition of our study group. Although data were collected across three Canadian provinces, from both urban and rural communities, and included distinct Aboriginal groups (i.e., First Nation, Inuit, and Métis), national representation and ability to generalize across all Canadian Aboriginal groups were nonetheless limited. While initially this may appear a limitation of the present study, this criticism would be misguided. For example, in Canada, there are 11 major Aboriginal language families and 65 distinct dialects [56]. To expect that there is a singular, representative, and general Aboriginal group in Canada, to which all results would apply, only serves to perpetuate biases that all Aboriginal groups are the same. While there are significant similarities that might be related to alcohol misuse (e.g., discrimination, disruptions to family connections, geographic dislocation, and abject poverty), all Aboriginal groups have rich cultures and histories that are unique. 

Second, participants in the present study did not entirely consist of Aboriginal youth, and included at least 12% of student drinkers who were nonAboriginal (see Endnotes 3 and 4). However, the 10 participating schools were schools with high proportions of Aboriginal students, and the large majority of the study participants were Aboriginal. The decision to include all student drinkers regardless of ethnicity/race was consistent with the wishes of our community partners (see Endnote 3) and enhanced our ability to generalize the findings to a wide variety of students attending such schools in Canada.

Several potential limitations to the present study should be acknowledged, each of which suggests important avenues for future research. The study was cross-sectional in nature and lies in contrast with the time frame of the theoretical model, which implies unfolding of relations between the study variables over time (i.e., hopelessness leading to the later development of depression, which leads to the eventual development of drinking to cope, which in turn results in excessive drinking). While this cross-sectional analysis is a first step in testing the utility of the proposed structural model, the model still requires further investigation within a multiwave longitudinal design [57]. Second, the proposed model is linear and unidirectional and does not acknowledge the possible reciprocal relations between study variables over time. For example, it is possible that excessive drinking actually increases depressive symptoms and/or hopelessness in the longer term either through the physiological or psychological consequences of heavy drinking [58]. It is also possible that depressive symptoms cause increases in hopelessness [59]. Such more complex reciprocal relations between study variables over time could be tested using longitudinal methods and multiwave data (e.g., see [60]), consistent with calls for examination of more complex models in Aboriginal alcohol research [61].

A third possible limitation is that study measures were developed for use with nonAboriginal adolescents and not all have been investigated in terms of their psychometric properties when used with Aboriginal youth. Nonetheless, all showed good internal consistency in the present study and some (e.g., DMQ-R coping motives subscale; [43]) have been previously validated in Aboriginal adolescents. Fourth, all study variables were assessed via retrospective self-report which may be subject to various biases including memory distortions and social desirability. Nevertheless, we did use methods for increasing the accuracy of participants' reports (e.g., [45]) and studies using other methodologies have shown similar results (e.g., sad mood induction leading to increased drinking in the lab among female young people who drink to cope; [62]). A forth potential limitation was that drinking to cope was assessed with a “generic” coping motives scale [39]. More recently, a measure has been developed and validated that distinguishes drinking to cope with* depression* from drinking to cope with *anxiety* [63]; this refined measure might be useful for future studies in this area.

Fifth, the present study did not consider potential moderators. For example, given known gender differences in depressive symptoms (greater in females [64]), drinking to cope with depression (greater in females [65]), and excessive drinking (greater in males [66]), future research should examine whether the hypothesized model is moderated by gender. Moreover, given recent evidence that resilience (individual, family, and community; [67]) buffers the effects of violence exposure on symptoms of posttraumatic stress disorder in Aboriginal youth [68], resilience might prove a useful variable to examine in future as a moderator in our proposed model. In particular, the construct of resilience might prove useful in further research to understand how some Aboriginal youth fare so well in terms of their emotional and behavioral health in spite of the gross social inequities they face daily in their environments. Given such a complex and multidimensional issue as excessive drinking among Aboriginal youth, it is likely that many environmental, interpersonal, and individual risk and protective factors will be found to play moderating roles in the preliminary model tested herein. Finally, the present study only focused on one possible pathway to excessive drinking in Aboriginal youth. While the present results do suggest that hopelessness may be one risk factor for excessive drinking in Aboriginal adolescents, other studies support additional risk pathways. For example, a recent study showed that exposure to violence was related to excessive drinking in Aboriginal youth and that this relation was mediated by symptoms of posttraumatic stress disorder rather than depressive symptoms [69]. 

The present findings of an indirect relation between hopelessness and excessive drinking suggest that targeted interventions for Aboriginal youth who are high in hopelessness are needed to prevent or decrease excessive drinking. The meditational results can be helpful for informing the content of such preventative or early interventions [70]. Mediational findings from the present study suggest the need to focus on depressive symptoms and maladaptive drinking to cope in targeted interventions for Aboriginal youth with high levels of hopelessness. Additionally, the unexpected direct path from hopelessness to risky drinking to cope suggests that hopeless cognitions may need to be a direct target in such preventative interventions as well. Cognitive practitioners would need to be particularly mindful of the objectively difficult circumstances facing many Canadian Aboriginal youth which set the stage for maladaptive, but not necessarily irrational, hopeless thinking styles. The direct effect of hopelessness on risky drinking to cope also points to the importance of primary prevention efforts (e.g., improving schools, developing sustainable local economies grounded in natural resources, and providing better education and employment opportunities) to deter the development of hopelessness among Aboriginal youth. A comprehensive approach to the problem of excessive drinking would pair community-wide primary prevention with school-based secondary prevention targeted toward high risk (e.g., high hopeless) youth. 

A cognitive-behavioral secondary prevention program focusing on hopelessness, depressive symptoms, and drinking to cope has been tested in the form of a school-based intervention among nonAboriginal youth via two randomized controlled trials (RCTs). The intervention targeting adolescents with high levels of hopelessness was shown to increase alcohol abstinence, decrease problem drinking [71], and reduce depressive symptoms [72]. More recently, this approach has been culturally adapted and has shown promise for reducing excessive and problematic drinking in a group of Canadian Aboriginal youth in an open trial [47]. The culturally adapted intervention still needs to be tested in an RCT. Such a trial could also test if intervention-induced changes in depressive symptoms, drinking to cope, and/or hopelessness mediate intervention-induced changes in excessive drinking among youth high in hopelessness at baseline. This would prove an even more stringent test of the theoretical model supported in the present study.

## 5. Conclusions

In sum, we used structural equation modeling to demonstrate the excellent fit of a model which links hopelessness to excessive drinking indirectly via depressive symptoms and drinking to cope in Canadian Aboriginal youth. Bootstrapping indicated that this indirect effect of hopelessness on excessive drinking was significant. Both depressive symptoms and drinking to cope should be intervention targets in school-based programs designed to prevent or decrease excessive drinking among Aboriginal youth with high levels of hopelessness.

## Figures and Tables

**Table 1:** Descriptive statistics and bivariate correlations between study measures.

	(1)	(2)	(3)	(4)	(5)	(6)	Mean (SD)
(1) Hopelessness	—						14.1 (4.0)
(2) Depressive symptoms	.42**	—					7.4 (5.9)
(3) Drinking to cope	.26**	.39**	—				2.1 (0.9)
(4) Drinking quantity	.12**	.02	.25**	—			3.4 (1.4)
(5) Drinking frequency	.14**	.13**	.30**	.49**	—		2.7 (1.1)
(6) Binge frinking	.13**	.10*	.30**	.56**	.73**	—	2.8 (1.0)

Note. Sample sizes vary from 486 to 531 due to missing data on various study measures.*P < .05. **P < .01.

**Table 2:** Bootstrap analyses of hypothesized indirect effects.

			Bootstrap estimates	
Hypothesized Indirect effect	Unstandardized indirect effect	Standardized indirect effect	SE for standardized indirect effect	95% confidence interval for standardized indirect effect (lower and upper)
Hopelessness to drinking to cope through depressive symptoms	.167	.143	.025	.094, .192*
Depressive symptoms to excessive drinking through drinking to cope	.019	.123	.025	.074, .173*
Hopelessness to excessive drinking^a^	.013	.059	.029	.002, .116*

Note. SE = bias-corrected standard error. ^a^indirect effect is based on all indirect paths. *Confidence intervals excluding zero are significant at *P* < .05.

## Endnotes

1. Aboriginal people can include First Nations, Inuit, and Métis peoples as recognized by the Constitution of Canada [73]. In this paper, we use the term Aboriginal to refer collectively to people from all three groups. Otherwise, we specify the particular Aboriginal/Indigenous group in question.
2. The “Other” Aboriginal category included youth who self-identified as belonging to the following Aboriginal groups: Nakota, Inuit, Nakota-Cree, Cree-Saulteaux, Saulteaux, Assiniboine, Sioux, Dene, Nakota-Assiniboine, Cree-Metis, and Ojibway-Black.
3. All students at the six participating Saskatchewan schools were invited to be involved in the research including a number of Caucasian youth and a few Black youth. Elders and school partners stressed the importance of including all students in the study in order to demonstrate a cultural value of connectedness and collaboration rather than risk marginalization and stigmatizing of certain groups by exclusion from the study. Three of the six Saskatchewan schools included students from First Nations communities encompassed within the provincial government education division; the three other Saskatchewan schools included students from First Nations communities governed within the educational jurisdiction of the Tribal Council. The respectful cooperation among school governance partners to implement this study is noteworthy.
4. This relatively high rate of missing data for the ethnicity/race item is likely due to the manner in which the question was structured. Students were provided five options which consisted of the five groups we expected to be most represented in the total group of students based on consultation with Elders and school partners (i.e., Cree, Ojibway, Metis, Oji-Cree, and Dakota). If students did not self-identify with one of these five groups, they were asked to specify their ethnicity/race in an open-ended item placed on the survey itself. It appears that many students either did not understand the instructions for this item or did not wish to provide this information to the investigators.
5. The first and second authors, both of whom are registered clinical psychologists, independently rated the BSI-DEP items for potential overlap with the hopelessness construct. Both raters agreed that there was only one BSI-DEP item that could be considered redundant with hopelessness (i.e., BSI-DEP item 5 “I am hopeless about the future”). Our central analysis (i.e., the hypothesized structural model depicted in Figure 1) was rerun after rescoring the BSI-DEP with this overlapping item removed. We found our results were virtually unaltered when using a version of the BSI-DEP that dropped this overlapping (redundant) item. Detailed results of this supplementary analysis are available from the first author upon request.
